# Supplementary material for: Subcutaneous Administration of Apolipoprotein J-Derived Mimetic Peptide d-[113–122]apoJ Improves LDL and HDL Function and Prevents Atherosclerosis in LDLR-KO Mice
Source: Biomolecules. 2020 May 29;10(6):829. doi: 10.3390/biom10060829 (PMC7356811; doi:10.3390/biom10060829)
Supplement: Supplementary file 1 [file biomolecules-10-00829-s001.pdf]

Subcutaneous Administration of Apolipoprotein J-derived Mimetic Peptide D-[113–122]apoJ Improves LDL and HDL Function and Prevents Atherosclerosis in LDLR-KO Mice

Andrea Rivas-Urbina, Anna Rull, Joile Aldana-Ramos, David Santos, Nuria Puig, Nuria Farre-Cabrerizo, Sonia Benitez, Antonio Perez, David de Gonzalo-Calvo, Joan Carles Escola-Gil, Josep Julve, Jordi Ordoñez-Llanos and Jose Luis Sanchez-Quesada

Supplementary materials

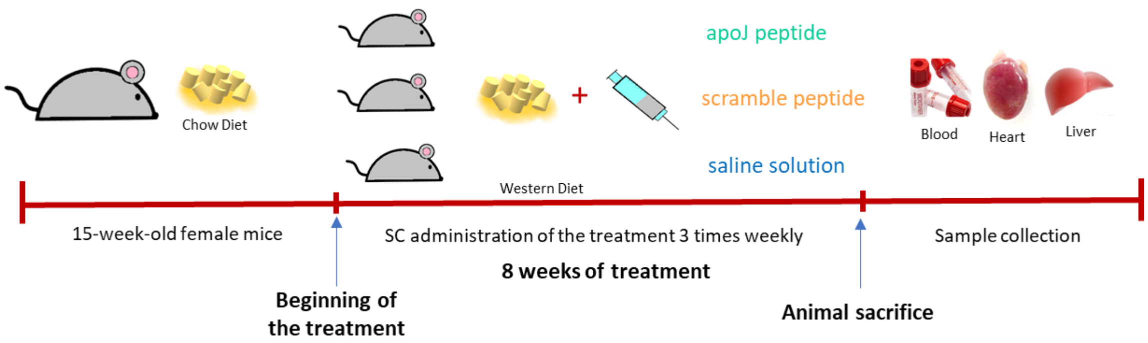

Figure S1. Experimental design flow chart.

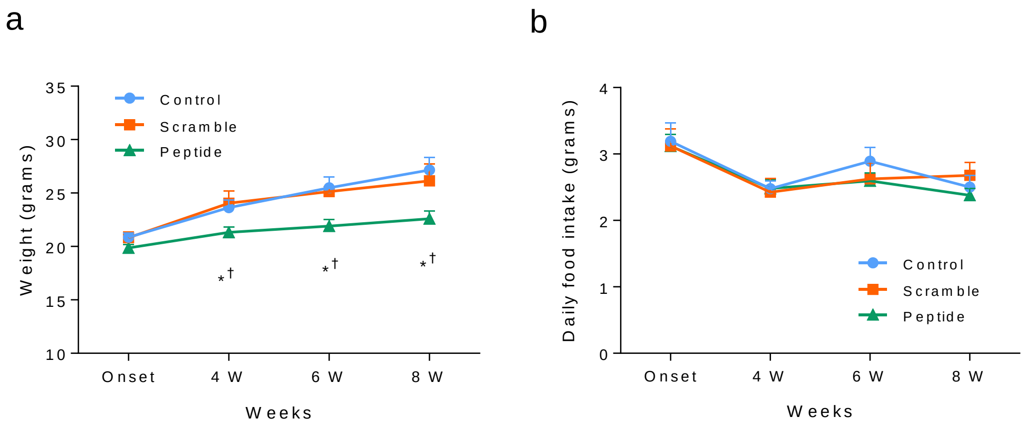

Figure S2. Weight-related parameters. (**a**) Increment of weight after 8 weeks on western diet. (**b**) Daily food intake. Data are the mean ± SEM of 8 mice from the control and scramble group, and 12 mice from the peptide group. * $p < 0.05$ vs control mice. † $p < 0.05$ vs scramble mice.

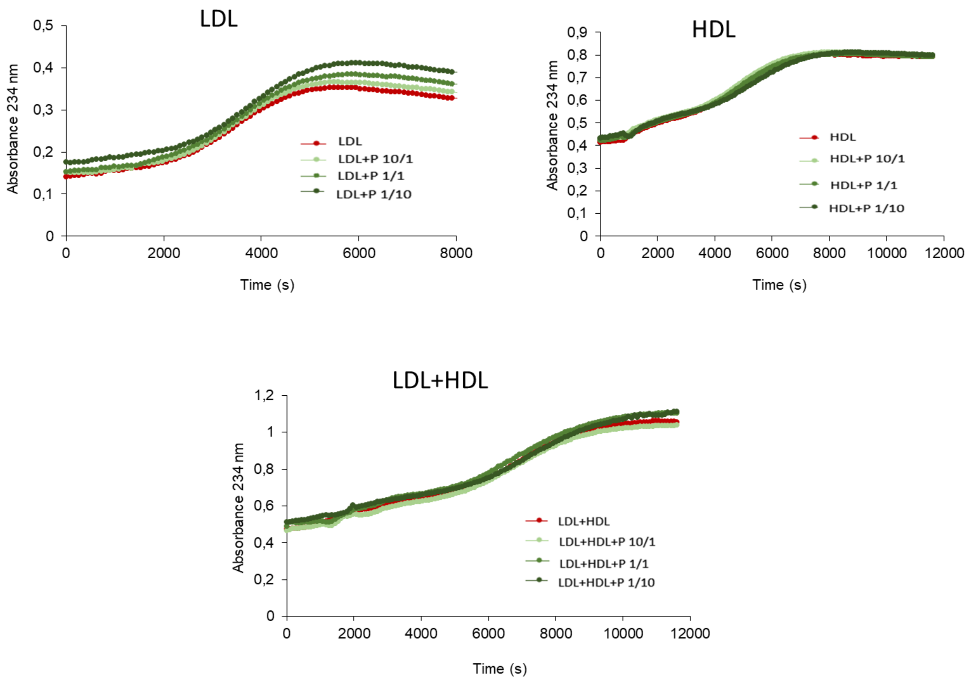

Figure S3. In vitro oxidation analysis of human LDL and HDL in presence of D-[113–122]apoJ peptide. Oxidation of lipoproteins was induced by adding 5 μM $CuSO_4$ and monitored at 234 nm, as described in Methods. Molar ratios of 10:1, 1:1 and 1:10 apoB:peptide or apoA-I:peptide were used.

Table S1. Univariate correlations of atherosclerosis development and lipoprotein function parameters. Data were analyzed using the Spearman's rho correlation analysis. Significant correlations are shown in yellow.

			Atherosclerosis	HDL_susceptibility_to_oxidation	HDL_Antioxidant_capacity	Cholesterol_efflux	LDL_susceptibility_to_oxidation	LDL_agreggation	LDL(-)
Spearman rho correlation	Atherosclerosis	Correlation coefficient	1,000	,656**	,300	-,460	,068	-,174	,730**
		Sig. (bilateral)		,005	,164	,098	,783	,427	,009
		N	24	24	24	14	24	24	12
	HDL_susceptibility_to_oxidation	Correlation coefficient		1,000	,452*	-,242	,660**	-,080	,011
		Sig. (bilateral)			,031	,404	,002	,716	,974
		N		24	24	14	24	24	12
	HDL_Antioxidant_capacity	Correlation coefficient			1,000	,185	,386	,271	-,105
		Sig. (bilateral)				,527	,102	,212	,745
		N			24	14	24	24	12
	Cholesterol_efflux	Correlation coefficient				1,000	-,096	,042	-,084
		Sig. (bilateral)					,754	,887	,830
		N				14	14	14	9
	LDL_susceptibility_to_oxidation	Correlation coefficient					1,000	,136	-,437
		Sig. (bilateral)						,578	,240
		N					24	24	9
	LDL_agreggation	Correlation coefficient						1,000	-,653*
		Sig. (bilateral)							,021
		N						24	12
	LDL(-)	Correlation coefficient							1,000
		Sig. (bilateral)							
		N							12

** P < 0,01 (bilateral).

* P < 0,05 (bilateral).

Table S2. Linear regression model relating atherosclerosis development with HDL susceptibility to oxidation and LDL electronegativity. Data are expressed as standardized beta (β).

**HDL Susceptibility to Oxidation**			**LDL(-)**		
Association between atherosclerosis and HDL susceptibility to oxidation after adjusting for each of the following variables (β):	β	*p*-Value	Association between atherosclerosis and LDL electronegativity after adjusting for each of the following variables (β):	β	*p*-Value
HDL Antioxidant capacity	0.618	0.004	HDL susceptibility to oxidation	0.577	0.011
Cholesterol efflux	0.562	0.036	HDL Antioxidant capacity	0.569	0.061
LDL susceptibility to oxidation	1.033	<0.001	Cholesterol efflux	0.486	0.210
LDL aggregation	0.611	0.002	LDL susceptibility to oxidation	0.681	0.088
LDL(-)	0.640	0.006	LDL aggregation	0.719	0.054
Cholesterol	0.634	0.005	Cholesterol	0.668	0.024
Triglycerides	0.628	0.002	Triglycerides	0.653	0.053
Phospholipids	0.620	0.002	Phospholipids	0.645	0.057
VLDL-c	0.637	0.002	VLDL-c	0.704	0.033
LDL-c	0.561	0.008	LDL-c	0.485	0.146
HDL-c	0.618	0.003	HDL-c	0.627	0.044

β estimates of HDL susceptibility to oxidation and LDL electronegativity when adjusted for each variable. Each row is a model containing that variable and the main variable in the heading.

Table S3. Association between atherosclerosis and treatment group after adjusting for the main variables of lipid profile and lipoprotein function. Data are expressed as standardized beta (β).

	**β**	***p*-Value**
Treatment group	0.455	0.029
Association between atherosclerosis and treatment group after adjusting for each of the following variables (β):		
HDL susceptibility to oxidation	0.320	0.070
HDL Antioxidant capacity	0.444	0.057
Cholesterol efflux	0.368	0.249
LDL susceptibility to oxidation	0.452	0.058
LDL aggregation	0.460	0.030
LDL(-)	0.257	0.415
Cholesterol	0.464	0.023
Triglycerides	0.553	0.018
Phospholipids	0.481	0.030
VLDL-c	0.492	0.023
LDL-c	0.368	0.105
HDL-c	0.526	0.014

β estimates for the treatment when adjusted for each variable in the row.
